# First Annual Commencement of the Training-School for Insane Asylum Attendants

**Published:** 1886-05

**Authors:** 


### First Annual Commencement of the Training-School for Insane Asylum Attendants.

A new era of reform in the treatment of the insane in our public institutions has been inaugurated by the medical officers of the Buffalo State Insane Asylum. An experience of many years with this unfortunate class of patients and the management of institutions for their treatment, convinced Dr. Judson B. Andrews that much practical benefit could be derived from the systematic training of attendants. No one will deny that the trained nurses in our hospitals are much superior to the self-educated (?) Mrs. Gamps of former times, whose knowledge was supposed to be derived from experience with the sick, but which often consisted of a lot of superstitious nonsense handed down by tradition. The nurse for the insane was also educated by experience, but she always had the advantage of intelligent guidance from the physicians in charge. Some who were watchful and attentive in their observations and duties became skillful attendants, but only after long years of service. In some institutions for the insane the important mental and moral qualifications, which every attendant should possess, were entirely disregarded. We well remember our experience with the muscular Pats and Bridgets, whose chief recommendation was their brawn. Such attendants so called will use physical force, and even cruelty, where a skillful nurse would, with little difficulty, manage their patients with a little persuasive kindness.

In order to obtain a competent corps of attendants for the Buffalo State Asylum, Dr. Andrews established, in October, 1883, "The Training School for Asylum Attendants," the first school of its kind in the United States. He was ably seconded in his efforts by his assistants, Drs. Granger and Crego, on whom most of the work of lecturing devolved. A graded course of study, extending over a period of three years, was arranged. A preliminary examination was required, and examinations for promotion were held at the end of each yearly course of study. The final examination was conducted by Dr. John Boardman, Dr. J. D. Hill, and Francis H. Root, trustees of the Asylum. Seven candidates passed this examination, Misses Phœbe Owens, Dora Hobson, Nellie Leitch, Anna Mahn, Margaret Lewis, Anna Blumenthal, and Clara Heppner.

The diplomas were presented by Mr. Francis H. Root, and the address to the graduating class was given by Dr. Stephen Smith, the State Commissioner in Lunacy. After giving a brief history of the methods of treating the insane in the past, Dr. Smith spoke of the competency of the force of attendants employed in our State Asylums at the present day. He congratulated the Buffalo State Asylum on being the pioneer in training attendants, and thought that the method would soon be adopted by other similar institutions.

It is impossible to bestow too much praise on Drs. Andrews and Granger for this important innovation, and we sincerely hope that the school may continue its prosperous course until it is able to number male as well as female trained attendants among its graduates.

