# Correction

**Published:** 1886-06

**Authors:** 


CORRECTION.

In our last number, in speaking of the training school for attendants at Buffalo State Asylum, we failed to state that the work of instructing the attendants in the rules of the institution and in emergencies has been carried on for a year by the present able second assistant physician, Dr. Hurd. This part of the work is important, and, at the same time, arduous. The manner in which Dr. Hurd is said by his chief officer to conduct this work is creditable alike to him and to the institution.

---

For want of space, we are unable to print an interesting account of the meeting of the American Medical Association; and of the meeting, in this city, of the Western Branch of Medical Association of the State of New York.

